# Global Warming in Pakistan and Its Impact on Public Health as Viewed Through a Health Equity Lens

**DOI:** 10.1177/27551938231154467

**Published:** 2023-02-02

**Authors:** Rozina Somani

**Affiliations:** 1 Faculty of Nursing, 7938 University of Toronto, Toronto, Ontario, Canada

**Keywords:** global warming, Pakistan, public health, flooding, climate justice, global health

## Abstract

Pakistan is extremely vulnerable to the negative impacts of climate change. The recent monsoon season caused widespread, deadly flooding, affecting 15% of the total population when extreme heat waves were followed by the worst rains and floods in the country's history. But Pakistan was not the cause of its own misfortune. The atmospheric buildup of carbon dioxide (CO2) is the greatest contributor to climate change. If we look at the increase of carbon dioxide in the atmosphere, we find that Pakistan is, like all developing nations, essentially a non-contributor of the problem, contributing considerably less than 1% of global greenhouse gas emissions. Moreover, although significant factors exacerbating the effects of climate change in Pakistan include an inadequate sewage system, air pollution from industrial waste, and deforestation, the country could not afford to proactively fix these, nor prepare for flooding and heavy rains. It lacks the funding for climate resilience efforts. As a result, Pakistan is suffering from a high prevalence of poor health outcomes. Children, the elderly, women, and the homeless, especially those living with poverty and disease, are at a high risk of morbidity and mortality. Since mitigating the devastating effects of climate change will continue to be an ongoing challenge for Pakistan, it urgently needs financial investment so that it can build climate-resilient infrastructures and institute mechanisms to deal with global warming's worst effects. Industrialized nations are responsible for global warming, and they must take responsibility for fighting global warming by helping developing countries cultivate greater public health emergency preparedness.

Global warming is a devastating and accelerating environmental public health problem that impacts every country around the globe. Significantly, however, this impact is uneven. Countries that have the fewest resources with which to combat global warming, and simultaneously suffer an increased health burden as a result of climate change, are experiencing some of the worst impacts of global warming.^1^ Among these, Pakistan is a prime example. In 2022, the temperature exceeded 122°F (50°C) in many regions of Pakistan.^2^ This increase in temperature during the monsoon season caused rains to be even heavier than usual in many parts of the country.^3^ As a result, Pakistan has been battered by flooding, which is affecting almost two-thirds of the country's districts. This has occurred despite having some warning: The Global Change Impact Studies Center in Islamabad cautioned that extremely high temperatures would result in heavy rains from July to September.^4^ Pakistan could not proactively prepare for flooding and heavy rains, because the government lacked the funding and climate resilience efforts to do so.^5^ Similarly, the construction of dams is one significant way to prevent floods, but Pakistan lacks the financial resources to build dams, as well as consensus on a national level on the types of dams, large or small, that would cater to the needs of all provinces without depriving them of water resources in the non-flooding season.^6^ As a result, widespread suffering has occurred in Pakistan. The Consumer News Business Channel (CNBC) reported that, because of the floods in Pakistan, more than 1,300 people have died and almost 1.2 million houses have been washed away. Moreover, crops, roads, schools, hospitals, and bridges have been destroyed across the country.^7^ According to the World Health Organization (WHO), more than 33 million people (15% of the total Pakistani population) have been affected by flooding. Within this group, approximately 6.4 million people are in such dire straits that they require humanitarian support. Although 634,000 people have been moved into temporary shelters created by the government and nongovernment organizations, they are still facing serious problems. In the relief camps, people are fighting with infectious diseases, including diarrhea, skin infections, and eye diseases.^8^

According to the Global Climate Risk Index, Pakistan is one of the most vulnerable countries to global warming and extreme weather events.^9^ Flooding, a significant problem, has several contributing factors. In the mountainous north, melting glaciers have caused high water flow into the Indus, Pakistan's largest river, resulting in a hazardous rush of water.^10^ Indeed, the Hindu Kush in northern Pakistan, known as “the third pole,” is among the regions most devastated by global warming.^11^ In addition, low-pressure heat waves in the Arabian sea contribute to heavy rains in the coastal provinces. As a result, this year Pakistan has seen a three- to fivefold increase in the normal amount of rain. Combined with accelerated glacial melt, this has led to devastating floods in many regions.^12^ Moreover, other significant factors—such as air pollution from industrial and agricultural waste and vehicles, an underdeveloped sewage system, limited freshwater resources, deforestation, and soil erosion—increase the threat when flooding does occur.^13^ Adding to these problems, Pakistan is far behind in implementing modern ecological stabilization technologies nor does it have the resources to do so, even if it were a priority. Both the industrialized West and Pakistan need to reevaluate, refocus, accept responsibility, and take action.^14^

Pakistan appears to be a recipient, rather than an instigator, of severe climate change. The atmospheric buildup of carbon dioxide (CO2) is the greatest contributor to climate change. However, Pakistan, like all developing nations, is, essentially, a noncontributor of the problem.^15^ If we look at the increase of carbon dioxide in the atmosphere between 1750 and 2020, we find that four countries have contributed more than 50% of the CO2 that creates global warming. The United States at 24.5% and China at 13.9% are distantly followed by Russia at 6.8% and Germany at 5.4%. In fact, historically, 15 countries have contributed about 75% of the increase in CO2.^16^ Today the picture is somewhat changed: China contributes 28% of the yearly global increase in CO2, more than twice as much as the United States at 13%. By contrast, Pakistan contributes considerably less than 1% of global greenhouse gas emissions.^17^

United Nations Secretary-General António Guterres said in a BBC article on August 30, 2022, that South Asian countries are more vulnerable to global warming. Indeed, he called them a “climate crisis hotspot” and pointed out that citizens of these countries are 15 times more likely to die from global warming emergencies. Guterres cautioned the world that curbing emissions was an urgent concern, exhorting people to “stop sleepwalking towards the destruction of our planet by global warming. Today, it's Pakistan. Tomorrow, it could be your country.”^18^ However, wealthy and developed countries don't just need to curb their emissions; they also need to take responsibility for funding and technically supporting Pakistan, while it develops strategies and implements outcomes to address climate change.

As a populous (242 million), low-income, developing nation and a former British colony that only regained independence in the mid-20th century, Pakistan, too, has issues around prioritizing and implementing an effective response to climate change. Political instability is probably the most fundamental issue. Pakistan has been, directly or indirectly, a military dictatorship for 30 out of the 74 years since independence.^19^ The main driver behind the political instability, as highlighted by Wasim,^20^ is that the state has many challenges to its autonomy and economy, including a lack of strong institutions, lack of accountability of leaders, and inadequate legislation to run the whole system. Added to political instability, Pakistan has severe resource constraints. The per capita income is approximately US$1,500, which is approximately 3% of Canadian per capita income. Even when adjusted to reflect purchasing power, the average Pakistani exists on only 10% of what the average Canadian receives.^21^ Due to the lack of accountability when taxes are unpaid, compounded by the absence of a standardized collection system, the country has, so far, been unable to create a structured tax collection mechanism. A huge gap exists between tax collection and gross domestic product (GDP) and between exports and GDP.^22^ Making matters worse, military spending, depending on how it is assessed, consumes 4% of GDP or 17% of government spending.^23^ Meanwhile, only 20% of the population has access to clean water, with 80% dependent on polluted water, causing high morbidity and mortality rates.^24^ Reforestation is another huge challenge for Pakistan. The United Nations assesses Pakistan's forest coverage at only 5%. This is low compared to the global average of approximately 31%.^25^ In addition, Pakistan and India, although neighbors, seem to prefer military competition to civil cooperation; this also damages the country's economy.^26^

Because of global warming's impact, Pakistan is suffering a high prevalence of poor health outcomes caused by heavy rains and floods. Children, the elderly, women, and homeless individuals, especially those living with poverty and disease, are at a higher risk of morbidity and mortality.^27^ Findings of a recent qualitative study conducted in five flood-affected districts of Pakistan indicate that the health system is inadequate, and therefore, unable to provide basic health facilities to flood victims in the affected districts.^28^

Currently, millions of internally displaced people are facing intense health challenges. The CNBC^7^ reports that millions of children are facing malnutrition, while approximately 73,000 women who are pregnant and expected to deliver soon do not necessarily have access to health care resources for their babies or postpartum care for themselves. Approximately 1,460 health facilities were damaged or destroyed during the flooding, and access to health care facilities is a huge issue for these internally displaced people. The WHO's representative to Pakistan, Dr. Palitha Mahipala, stated that the WHO is aiming to provide access to immediate health services for people living in camps. In addition to this, the WHO's focus is surveillance in flood-affected regions to control disease outbreaks.^8^ Similarly, national and international organizations such as WHO, UNICEF, and the Aga Khan Development Network have initiated flood relief campaigns and provided resources and funding so the country can manage the aftereffects of this hazardous episode of global warming. However, rather than simply rebuilding the country's infrastructure, Pakistan urgently needs financial investment so that it can build climate-resilient infrastructure and institute mechanisms to deal with global warming and its future effects. This financial investment should come from wealthier countries, which are responsible for the effects of global warming, rather than countries such as Pakistan.^29^

To conclude, this year Pakistan is facing an unprecedented health burden. The country is still fighting COVID-19, and the current flooding has created a host of serious health challenges for the population. In affected areas, people are at high risk for many waterborne, vector-borne, and infectious diseases. Flood-related mortality and morbidity rates are continuously rising in the country.^30^ The country's economy, which was already floundering, is now seriously threatened by the destruction caused by flooding and the subsequent disease burden. Many national and international organizations are coming forward to support the victims by generating funds; creating shelters; and providing food, drinking water, and health access. Still, many people are suffering badly due to the absence of resources. Pakistan needs major support from wealthy countries and international organizations so that it can overcome this disastrous situation. Industrialized countries should take responsibility for fighting global warming by helping developing countries cultivate greater public health emergency preparedness. The world must not sit idly by and wait to see whether the same sort of flooding occurs next year. That would be catastrophic for 240 million Pakistanis. Urgent action is needed.
